# Squamous cell carcinoma in situ and syringocystadenocarcinoma papilliferum overlap in an Asian patient: A case report and review of the literature

**DOI:** 10.1016/j.jdcr.2026.07.044

**Published:** 2026-07-31

**Authors:** Xer Min Nicole Lau, Po Yin Tang, Kok Hing Lim, Choon Chiat Oh

**Affiliations:** a Department of Dermatology, Singapore General Hospital, Singapore, Singapore; b Division of Pathology, Singapore General Hospital, Singapore, Singapore; c Duke-NUS Medical School, Singapore, Singapore

**Keywords:** adnexal tumor, Asian, squamous cell carcinoma, syringocystadenocarcinoma papilliferum

## Introduction

Syringocystadenocarcinoma papilliferum (SCACP) is a rare neoplasm arising from apocrine glands and is regarded as a malignant counterpart of syringocystadenoma papilliferum. Most cases are believed to arise via malignant transformation of syringocystadenoma papilliferum, whereas some have been reported to be associated with nevus sebaceous through multistep progression^1^ or even de novo.^2^

Clinically, it presents as a flesh-colored to hyperpigmented exophytic nodule or inflammatory plaque, often accompanied by ulcerations and secretions. Its characteristic crust is attributed to decapitation secretion from apocrine glands.^3^ In view of its rarity and variable presentation, SCACP may clinically be mistaken for other cutaneous malignancies, including squamous cell carcinoma (SCC).^4^

We present a rare case of SCC in situ with underlying SCACP over the left forearm in an Asian patient, which is the fifth reported case to date.

## Case report

A 73-year-old Chinese man with a history of systemic lupus erythematosus with Evans syndrome and left upper lobe lung SCC and Bowen disease of the left long finger presented to the dermatology clinic for a persistent nonhealing ulcer on the left forearm for 1 year. There was no fever, tenderness, or pruritus in the affected area. There were no previous surgeries or prior trauma to site. Physical examination showed a 2.4 × 2 cm left forearm plaque (Fig 1). Regional lymph nodes were not palpable.

**Fig 1:**
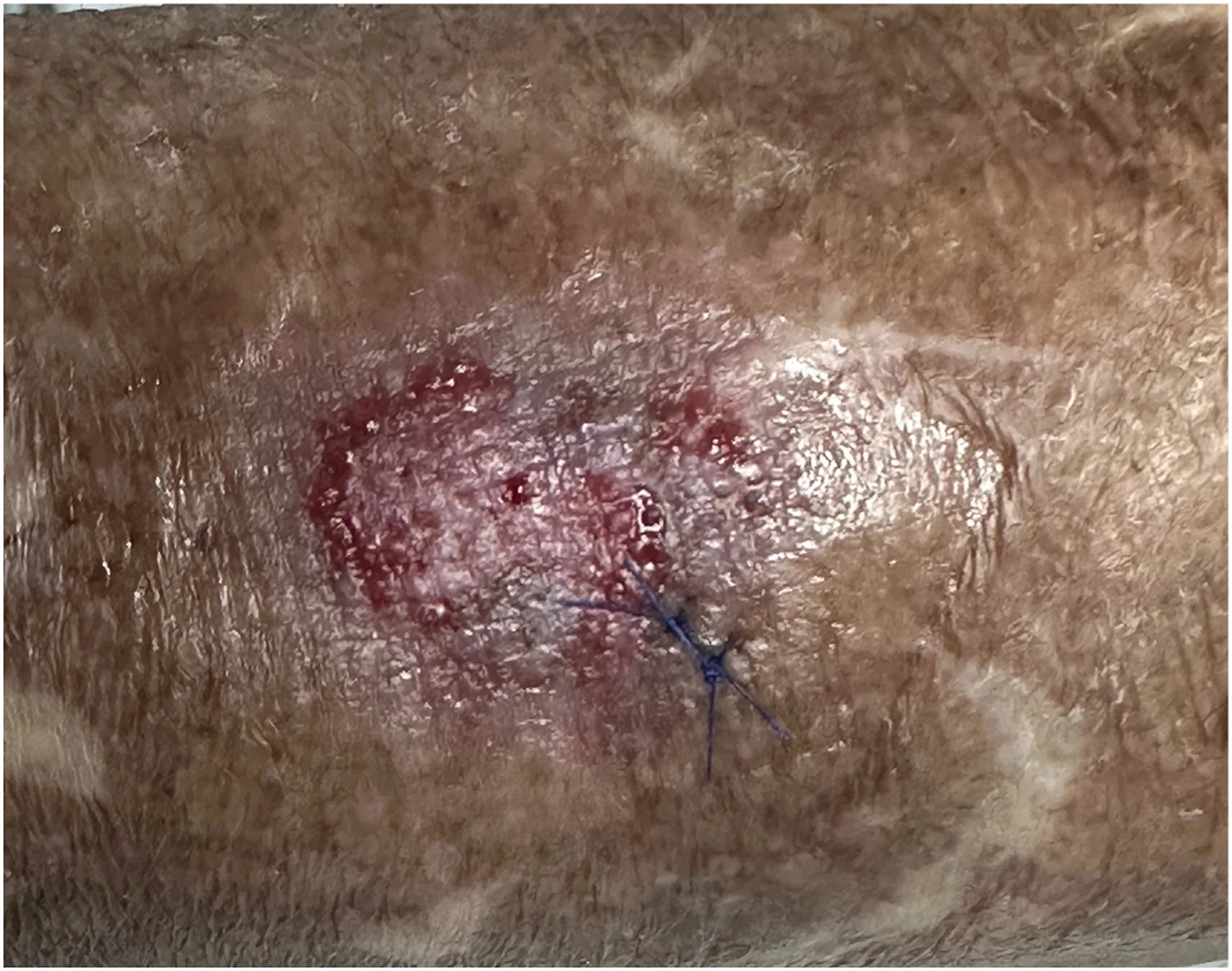
Clinical photographs of a 2.4 × 2 cm left forearm plaque.

Initial incisional biopsy demonstrated features consistent with SCC in situ, characterized by irregularly acanthotic epidermis with near-to-focal full-thickness squamous dysplasia, extending to one edge of biopsy (Fig 2). Immunostaining showed strong p40 positivity, weak and patchy positivity for epithelial membrane antigen, and negativity for Sry-related HMg-Box gene 10, Bologna epithelial - epithelial protein 4, and Anti-Cytokeratin 5.2 (Supplementary Figs 1 and 2, available via Mendeley at https://data.mendeley.com/drafts/p9dstr9hpm).

**Fig 2:**
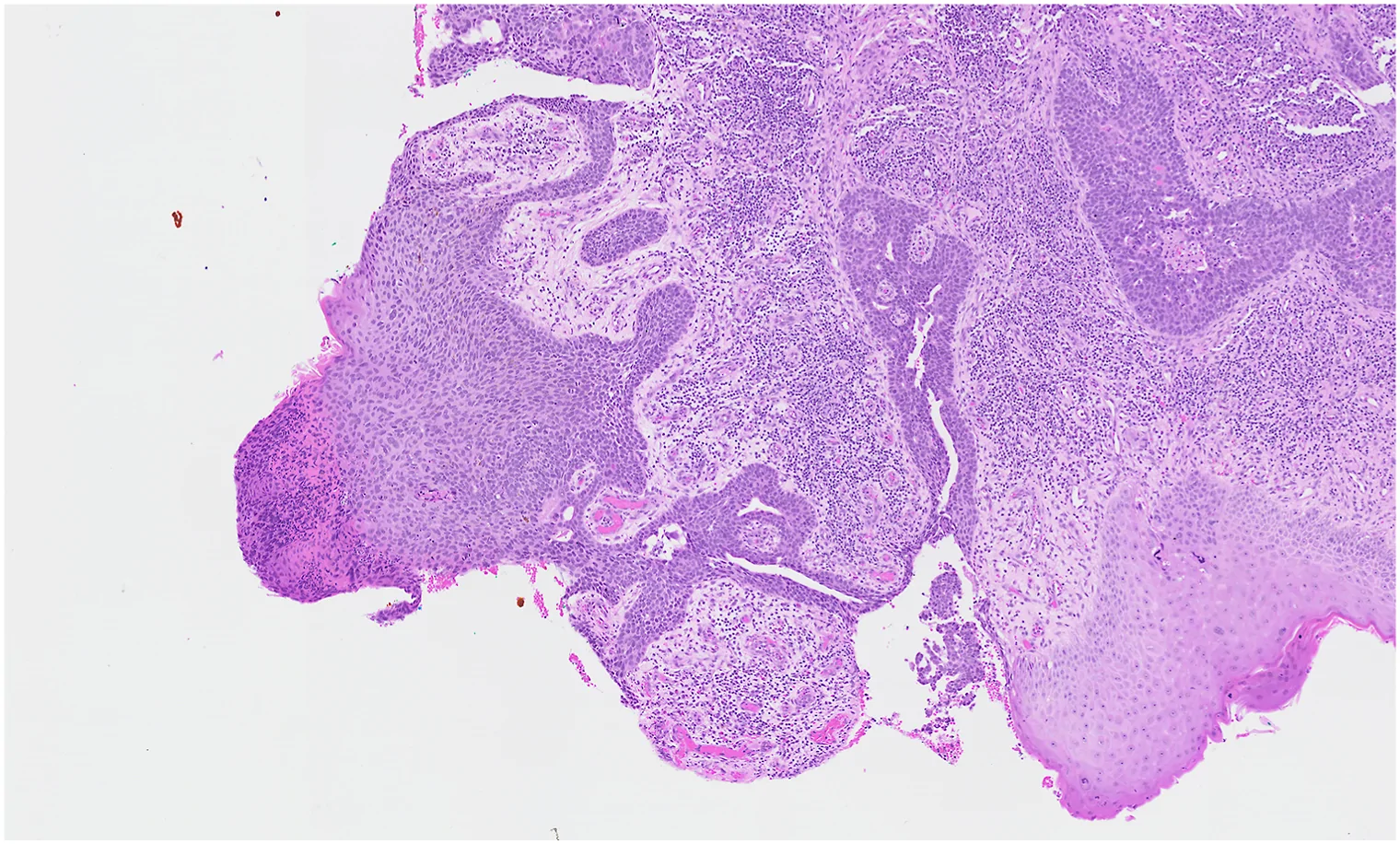
High-grade squamous dysplasia is seen with extension into dermal adnexal structures. Dermal adnexal structures also appear to form invaginations and show papillary formations with fibrovascular cores and micropapillary projections, raising the possibility of an underlying syringocystadenoma papilliferum colonized by squamous dysplasia.

Complete excision of the lesion was subsequently performed. In addition to the previously identified SCC in situ, further sections revealed underlying invaginated epithelial nests extending into the dermis. These formed papillary and micropapillary structures composed of markedly atypical epithelial cells, with identifiable foci of dermal invasion (Fig 3).

**Fig 3:**
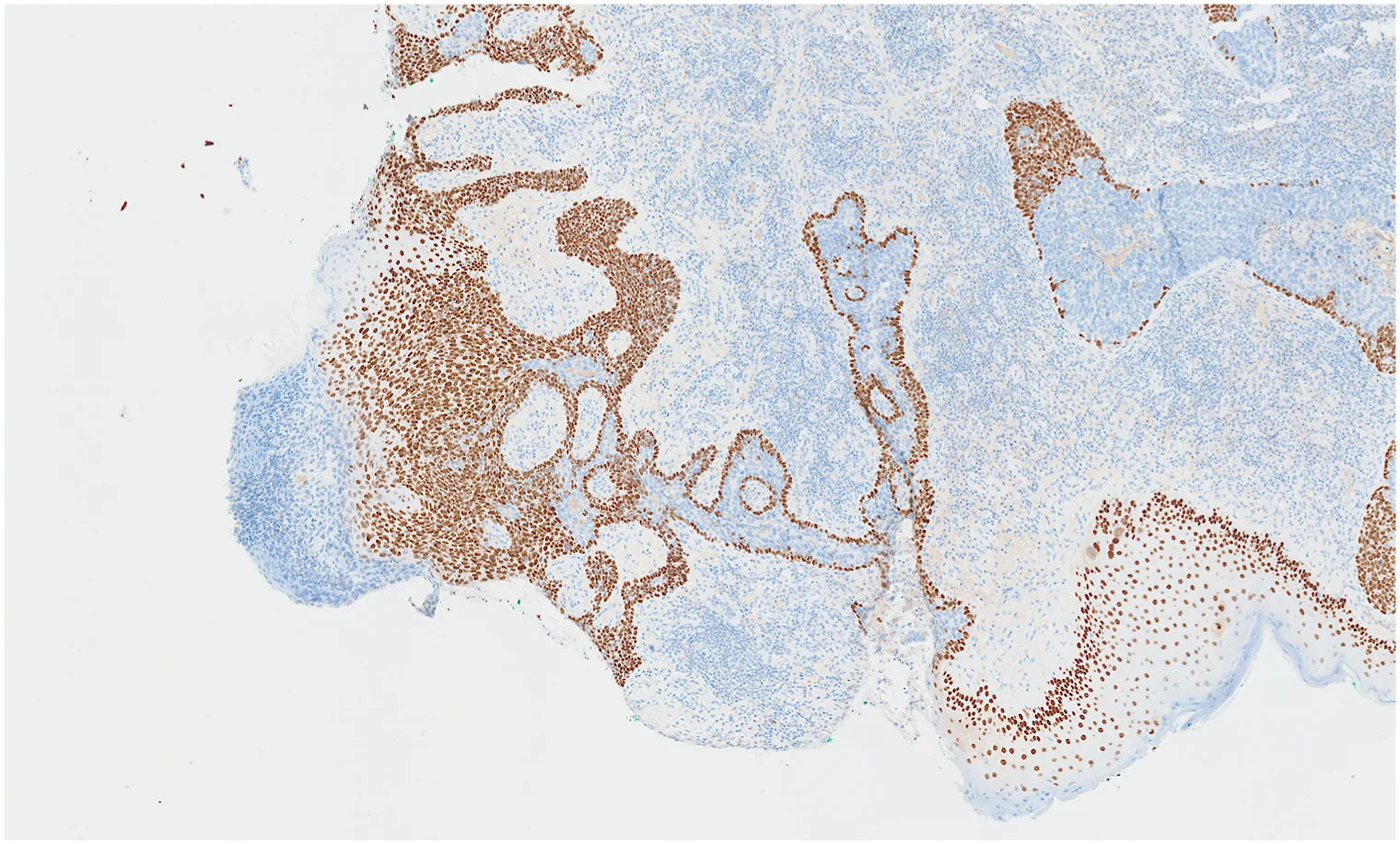
P40 staining is positive in squamous cells and demonstrates retention of basal layers, favoring against any invasive carcinoma.

Immunohistochemically, the epidermal dysplastic component showed strong p40 expression and was negative for Bologna epithelial - epithelial protein 4 (Fig 3). In contrast, the dermal neoplastic component demonstrated the opposite staining pattern, supporting the presence of 2 distinct but contiguous neoplastic processes (Fig. 4). The surface dysplasia showed comparatively stronger GATA-binding protein 3 uptake with some focal androgen receptor and Sry-related HMg-Box gene 10 positivity. Both components were negative for synaptophysin, Nuclear Protein in Testis, and calretinin. Overall findings supported a diagnosis of SCACP with overlying SCC in situ.

**Fig 4:**
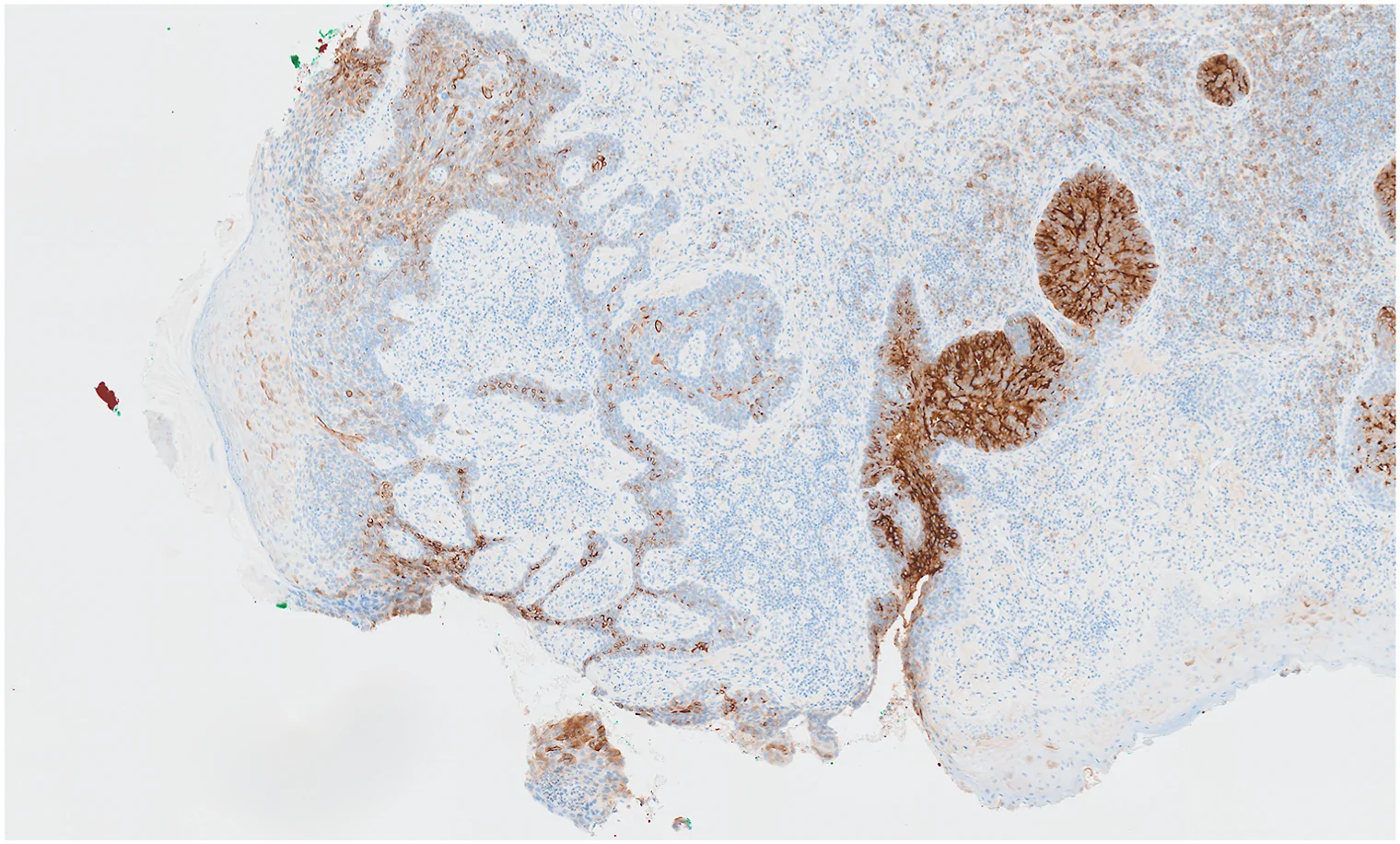
Epithelial membrane antigen staining shows residual luminal cells within the dermal lesion.

In view of the clinicopathologic correlation of SCC in situ with concomitant dermal SCACP, a wide surgical excision was offered for margin clearance; however, the patient declined reexcision and accepted risk of underlying malignancy and disease progression. There was no local recurrence of the tumor at 6-month follow-up.

## Discussion

### Reported cases and epidemiology

• A literature review of Cochrane and PubMed identified 48 cases of SCACP, excluding foreign language articles.
• Predilection for older adults seen; the median age was 64 years (range, 26-93 years).
• A slight male predominance with 28 men (58.3%) and 20 women (41.7%).
• Most frequently involved site was the head and neck region, particularly the scalp (*n* = 16, 33.3%), followed by the perianal and chest regions (*n* = 5, 10.0% and *n* = 4, 8.3%, respectively), with only 2 cases reported over the upper limb.

### Metastatic potential and histologic features

• Among the cases with documented metastatic status (*n* = 14)
  o 5 of 14 had regional lymph node involvement
  o 3 of 14 with distant metastasis
  o Prior literature reviews have reported metastatic progression of up to 17%.^2^
• Histologically, SCACP retains architectural features of syringocystadenoma papilliferum, including cystic, papillomatous invaginations extending from the epidermis to the dermis, connected by funnel-shaped structures lined with infundibular epithelium. The stroma of the tumor consists of a dense inflammatory infiltrate of plasma cells and lymphocytes.^5^ However, SCACP exhibits higher nuclear and cellular atypia, increased mitotic activity,^6^ and an infiltrative adenocarcinomatous component.^2^ Histopathologic diagnosis may pose as a challenge because of the resemblance to other adnexal tumors such as hidradenoma papilliferum or cutaneous metastasis of malignancies from breast, gastrointestinal cancers, or thyroid carcinoma.^7^

### Diagnostic challenges

• Immunohistochemical findings in SCACP are variable; expression of markers such as p63 supports an adnexal origin and assists in excluding metastatic disease.^8^

### Coexistence with SCC

• Coexistence of SCACP with SCC is exceedingly rare, with only 4 cases reported to date (Table I).

**Table I:** Syringocystadenocarcinoma with concomitant squamous cell carcinoma

Authors/publication year	Age/race (if specified)	Presentation	Treatment/outcomes
Arslan et al (2013)^9^	66-y-old male	Parietal aspect of the left side of the scalp Present for 20 y before ulceration appeared in the last 2 y	Presence of regional metastases of SCACP in 3 nodes of left cervical region (15-mo follow-up) Excised with 2 cm surgical margins Radiation therapy 2 mo after surgical excision
Arslan et al (2013)^9^	66-y-old female	Frontotemporal aspect of the right side of the region Present for >1 y	No signs of recurrence 2 y after surgery Wide excision with 2 cm surgical margins
Yan Zhang et al (2017)^10^	63-y-old male Chinese	Axilla Present for 3 mo	NA
Yan Zhang et al (2017)^10^	29-y-old female Chinese	Forehead Present for 2 y	NA

*NA*, not available; *SCACP*, syringocystadenocarcinoma papilliferum.
• The presence of SCC may obscure the underlying adnexal malignancy, posing a diagnostic pitfall, especially in superficial biopsies. Recognition of this is crucial given that SCACP carries risk of local recurrence and metastasis that differs from that of SCC alone.
• Prognosis of these concomitant cases has been poorly studied given the rarity of such an occurrence.

### Treatment and prognosis

• SCACP is generally a low-grade malignancy with relatively favorable prognosis following complete surgical excision (Supplementary Table I, available via Mendeley at https://data.mendeley.com/drafts/p9dstr9hpm).
• Locoregional metastasis is reported in a minority of cases, and systemic therapy is not routinely indicated in absence of advanced disease.^11^
• Prolonged surveillance is recommended, as recurrences as late as 8 years after excision^10^ have been documented.
• Wide local excision with histologically clear margins is the main modality of treatment. Mohs micrographic surgery may be considered, although comparative outcome data are limited. The role of adjuvant radiotherapy remains uncertain in efficacy because of the rarity of the malignancy.
• Given the rare coexistence of SCC in situ and SCACP in our patient, judicious long-term follow-up is warranted.

## Conflict of interest

None disclosed.
